# Challenges of Screening and Investigations of Contacts of Patients with Tuberculosis in Oyo and Osun States, Nigeria

**DOI:** 10.3390/tropicalmed9070144

**Published:** 2024-06-28

**Authors:** Aderonke Agbaje, Patrick Dakum, Olugbenga Daniel, Anyaike Chukwuma, Obioma Chijoke-Akaniro, Evaezi Okpokoro, Samuel Akingbesote, Christian Anyomi, Adekola Adekunle, Abiola Alege, Moroof Gbadamosi, Olutunde Babalola, Charles Mensah, Rupert Eneogu, Austin Ihesie, Ademola Adelekan

**Affiliations:** 1 Department of Prevention, Care and Treatment, Institute of Human Virology Nigeria, Abuja 900231, Nigeria; aagbaje@ihvnigeria.org (A.A.); pdakum@ihvnigeria.org (P.D.); odaniel@ihvnigeria.org (O.D.); eokpokoro@ihvnigeria.org (E.O.); sakingbesote@ihvnigeria.org (S.A.); canyomi@cihpng.org (C.A.); cmensah@ihvnigeria.org (C.M.); 2 Department of Public Health, National Tuberculosis, Leprosy and Buruli Ulcer Control Programme, Federal Ministry of Health, Abuja 900211, Nigeria; chuxxanyaike@yahoo.com (A.C.); ockaniro@gmail.com (O.C.-A.); 3 Department of Prevention, Care, and Treatment, Society for Family Health, Abuja 900247, Nigeria; aalege@sfhnigeria.org; 4 Department of Public Health, Osun State Ministry of Health, Osogbo 230284, Nigeria; gbadamosideji@yahoo.com; 5 Department of Public Health, Oyo State Ministry of Health, Ibadan 200214, Nigeria; sumjohnbabloo68@yahoo.com; 6 HIV, AIDS, and TB Unit, United States Agency for International Development, Abuja 900211, Nigeria; reneogu@usaid.gov (R.E.); aihesie@usaid.gov (A.I.)

**Keywords:** tuberculosis, contact investigation, challenges, existing gaps

## Abstract

Tuberculosis (TB) remains a significant public health challenge in Nigeria, with high rates of transmission and low case detection rates. This paper presents the challenges of screening and investigation of contacts of patients with TB in Oyo and Osun State, Nigeria. This descriptive-qualitative study was conducted in eight Local Government Areas with high TB burdens. Twenty-four focus group discussions and 30 key informant interviews were conducted among TB patients, household TB contacts, and government TB staff, among others. Respondents ages ranged from 17–85 years with a mean of 42.08 ± 14.9 years, and (4.0%) had a postgraduate degree. This study identified that the majority of TB contacts who tested negative for TB were unwilling to be placed on TB preventive therapy because of the belief that only a sick person should take drugs. Also, hostility from the TB contacts to the contact tracers during the house-to-house screening of presumptive TB cases due to community stigma associated with TB was another existing gap reported in TB contact investigations. The findings emphasise the importance of tailored approaches in TB prevention and control, addressing challenges in testing and contact investigations; this necessitates investments in community engagement strategies to enhance the cooperation of TB contacts.

## 1. Introduction

Tuberculosis (TB) remains a significant public health problem in Nigeria, with a high burden of both active and latent TB cases. Worldwide, 10.6 million people (95% UI: 9.9–11.4 million) developed TB in 2022, up from best estimates of 10.3 million in 2021 and 10.0 million in 2020. Globally, the estimated TB incidence rate (new cases per 100,000 people per year) was 133 (95% UI: 124–143) in 2022. The net reduction from 2015 to 2022 was 8.7%, far from the WHO End TB Strategy milestone of a 50% reduction by 2025 [1].

Nigeria accounts for 4% of the global gap between new TB cases and notified cases (diagnosed, treated, and reported). Nigeria has a TB incidence rate of 219 per 100,000 people, with an estimated 467,000 people with active TB disease. In 2021, according to the National TB, Leprosy, and Buruli Ulcer Control Programme (NTBLCP), 207,785 were notified, which gave rise to a gap of 56% [2]. Untreated TB cases can infect 10 to 15 persons yearly; hence, undiagnosed cases raise the risk of TB transmission [3]. Therefore, identifying and notifying the missed cases is essential for efficient TB control. Contacts are defined as individuals who have spent prolonged periods (usually more than 8 h) in close proximity to the index case, such as family members, colleagues, or classmates [4].

Contact tracing is an essential component of TB control in Nigeria, as it helps to identify individuals who may have been exposed to TB and are at risk of developing the disease. Identifying index TB cases is crucial in initiating contact tracing [5]. Contact investigation is a key element of TB control in high-burden countries like Nigeria. However, there are several challenges to effective contact investigation in Nigeria, including limited resources, poor coordination among healthcare providers, and inadequate community engagement [6]. There are significant gaps in several areas of TB programming, such as TB contact investigations, despite continued attempts to control and eradicate TB. It is critical to develop an in-depth understanding of the challenges and opportunities encountered in the field to address these gaps and enhance TB control activities. According to Oshi et al. [7], community engagement and participation are crucial for effective TB contact investigation; however, community participation is often limited in Nigeria [8]. Limited healthcare access, particularly in remote and hard-to-reach areas, also poses a challenge to TB contact investigation in Nigeria [7].

Nigeria weak health system also has inadequate infrastructure, poor funding, and limited coordination among different health system levels. Many healthcare facilities have been reported to lack the necessary equipment, supplies, and personnel to conduct effective contact investigations [9]. By identifying the specific gaps and challenges in TB contact investigations, appropriate interventions and strategies can be developed to strengthen TB programming in the Oyo and Osun States. The findings of this research initiative will provide valuable information to inform policy and programmatic decisions aimed at enhancing TB control efforts in the two states. This study, therefore, aims to identify the challenges of screening and investigation of contacts of patients with TB in Oyo and Osun States, Nigeria.

## 2. Materials and Methods

### 2.1. Study Design

This descriptive qualitative study was used to ascertain challenges with TB screening and existing gaps in contact investigations in Oyo and Osun States, Nigeria.

### 2.2. Study Area

This research was carried out in eight (Osun = four; Oyo = four) Local Government Areas (LGAs) with a TB high burden in Oyo and Osun States, Nigeria. The LGAs are part of the United States Agency for International Development (USAID) supported facilities through the Tuberculosis Local Organizations Network Region 3 (TB LON 3) project being implemented by the Institute of Human Virology, Nigeria (IHVN). In Oyo State, the study LGAs were Ibadan North, Oyo East, Ogbomoso South, and Iseyin, while in Osun State, Iwo, Oshogbo, Ife Central, and Ede South were used for the study.

In Oyo and Osun States, contact tracing is done with the support of TB patients, who will be asked to invite their household contacts to the facility for screening. When the contacts refuse to come, the TB patients will provide their contact phone number, and either the DOTS officer or contact tracers will call and invite the household contacts to the facility for screening. When contacts refuse to come, a home visit will be scheduled for such contacts to be screened systematically for TB. Those identified as presumptive will be asked to produce a sputum sample, which will be subjected to Gexepert screening. Based on the symptomatic screening, those who are not presumptive will be placed on TB preventive therapy (TPT).

### 2.3. Target Population

The target population for this study were bacteriologically diagnosed pulmonary TB patients (TBP), household TB contacts (HTBC), and Community Volunteers (CV). Others were the Ward Development Committee (WDC) chair, government-employed TB personnel (Directly Observed Treatment, Short-course (DOTS) officers, Tuberculosis and Leprosy Supervisor (TBLS) officers, State Tuberculosis and Leprosy Managers (STBLM)), and TB LON 3 project staff. The TBP, HTBC, CV, and WDC Chair were invited to the health centres for the interviews, while TBLS, DOTS, STBLM, and TB-LON 3 staff interviews were done in their office.

### 2.4. Sample Size

The saturation method was used for this study; however, 24 (12/State) Focus Group Discussions (FGDs) were conducted, as well as 30 (15/State) Key Informant Interviews (KIIs). The participants for the FGDs were TBP, HTBC, and CVs. Each FGD group is made up of six to 10 participants. Also, KII were conducted among DOTS officers, TBLS officers, STBLM, WDC chair, and TB-LON 3 staff (Table A1). The choice of KII for the stakeholders was associated with their numbers, which could not form the minimum required number of participants to use for the FGD session. For instance, we have just one STBLM in each State and one TBLS in each LGA.

### 2.5. Sampling Techniques

The participants for this research were chosen using a four-stage sampling method.

**Stage 1:** Using the IHVN (Institute of Human Virology Nigeria) program sites, a purposeful sampling method was used to select two States (Oyo and Osun) in the South-West of Nigeria.

**State 2:** Four LGAs were randomly selected, each from Oyo and Osun States.

**Stage 3:** One facility was chosen from each LGA using random numbers generated from www.randomizer.org (accessed on 21 September 2022) from the IHVN-supported DOTS facilities list.

**Stage 4:** In the catchment areas of the chosen DOTS facilities, a purposeful sampling method was used to select all the stakeholders required for the study.

### 2.6. Instruments and Data Collection

The FGD and KII guides were used for data collection for this study. The FGD guide was used among TBP, HTBC, and CVs. On the other hand, the KII was used among DOTS officers, TBLS officers, STBLM, WDC Chair, and TB-LON 3 project staff. The Validated tools—KII and FGD guides—were developed based on literature review and consultation with public health and TB experts. The validation was done through an extensive literature search, a review from field experts, and the tools pre-test among the homogenous population in Ibadan. Translation to Yoruba and back translation was also done to ensure the instruments were suitable for the local context. The tools were used to collect information on the participants sociodemographic characteristics, challenges with screening, and investigations of contacts of patients with TB between November and December 2022. The FGD discussion lasted 60–90 min, while KII lasted 30–60 min. All the interviews were audio recorded with the consent of the participants.

### 2.7. Data Management and Analysis

The note-takers transcribed the audio recordings verbatim, and field notes were used to beef up audio recordings. The transcribed notes were further subjected to validation by three of the authors. Four authors then coded the data and reviewed it with others before major themes were first identified. Transcripts were also reviewed for themes through a continuous process of data segment comparison based on qualitative research techniques. A codebook was developed defining themes, and a numeric theme code was assigned to each particular category of text responses. The analysis was systematic and involved categorising data. Additionally, Nvivo 12 software was used to re-analyse.

## 3. Results

### 3.1. Respondents/Participants Sociodemographic Characteristics

The respondents ranged from 17 to 85 years, with a mean age of 48.6 ± 8.5 years and 40.4 ± 15.7 years among KII and FGD participants, respectively. More than half (75.6%) of the FGD participants were male, compared with 24.4% in KII. An equal proportion (50.0%) of KII and FGD participants had a tertiary education (Table A2).

### 3.2. Challenges with TB Screening

When asked about the challenges faced so far concerning TB screening, more than half of the government officials said insufficient diagnostic tools to cater for the number of samples generated via various demand-creation activities, low turn-over time of test results from within 24 h previously to an average of four days to six weeks currently. This, in turn, leads to the delay in the initiation of TB treatment and further enhances the circulation of TB disease and other comorbidities in the community. One of the STBLM Officials specifically stated that;

*“One of our key challenges is a grossly inadequate number of screening tools. I mean the gene expert that is meant for the test. Presently, we have 13 gene experts and other testing facilities, but it is not enough; it is grossly inadequate. As it is what we are having in this is that we do not get results within 24 h. The result often takes three, four, or six weeks, and you can imagine that when people get to laboratories and are not getting their results, you can imagine what will happen. So they will go back to the community. So, when people are not treated, the infection continues circulating in the community.”*

In addition, an inadequate number of trained TBLS in some local government areas was also mentioned as one of the challenges faced by some government officials. They further said that the country security volatility had created fear among the supervisors about travelling to Zaria for the training. It was, however, suggested that if the training could be held in Lagos or a sub-central state in the country, it would encourage more personnel to become trained TBLS in the State. Furthermore, there is an inadequate supply of reporting and recording tools, minimal human resources in the deployment of the different components of TB program activities, an insufficient supply of TB treatment drugs and HIV testing kits, non-availability of project vehicles for mobility to enable routine supportive supervision across the State were also listed as part of the challenges currently faced in the execution of the TB program activities. One of the STBLM Official stated that;

*“We are also not having adequate reporting and recording tools…these things are grossly inadequate…we do not have adequate HIV testing kits, and it is affecting us; we need to test every identified tuberculosis, whether they have a TB as well as HIV is very important…the other thing is HR, we have a very minimal number of human resources, we do not have enough DOT staffs in our facilities…we also need a project vehicle to be able to have supervision…so, we need a vehicle to move around from time to time every quarter to see that our patients are given all the standard drugs. So, these are the few critical challenges we have; if we have a solution, it goes a long way to improve the States.”*

One of the TBLS Officers from Oyo State also;

*“In terms of tracing patients, we have challenges. Before, they used to give us a motorcycle that we use to trace the patients in the community; they gave us everything we needed to maintain it, but now, we do not have such a thing, and they do not supply us with a motorcycle again...”*

The majority of the community volunteers listed the nonadherence of some TB cases to the drug dosage, refusal of some contacts of TB cases to getting screened for TB, and lack of funds for the TB cases to eat and visit the health facility for their drug refill as some of the challenges faced. A participant from Oyo State said;

*“Those that have TB are usually stubborn and completely disagree that they do not have TB; some will agree to start drugs that same day, and you know that drug works like magic when they start using drugs for two weeks, and they have been relieved, they tell us they are no longer using drugs, some will run away, we look for them, we will call them, some of them will start abusing us on the phone. They once told us they would beat us if we passed their area. It happened before when I started working, and that is one of our challenges.”*

According to the TB-LON 3 project staff, the low turn-around time of the test results due to the limited number of Gene Xpert machines in the State and the unwillingness of the males in the community to get screened for TB were listed as the significant challenges faced with TB programming. The need to provide more TB diagnostic machines and targeted TB sensitisation to the male gender was highlighted as a strategy that can help mitigate this challenge. One of the TB-LON 3 project Staff said;

*“When you get to the field, you will only see females showing up for TB screening. Now, let us put it that it is in the natural nature of men to hustle…they are out there, and they do not have time for this. Now, they can be the major carrier, or they can also be the ones to catch it some other time out there in the field; females tend to turn out more than males, so what can happen there is that whichever way the awareness should be done or sensitisation about male coming out whenever there is an outreach, I would encourage that.”*

Furthermore, the fact that the diagnostic capacity of the State has been currently overwhelmed by the increased number of samples generated via facility- and community-based screening and the outright refusal of some patients to be screened for TB were also listed as the challenges faced.

### 3.3. Investigation of Contacts of Patients with TB

Most of the government officials mentioned that the TB contacts are supportive of getting screened and tested for TB; however, the majority who tested negative for TB are unwilling to be placed on TB preventive therapy because of the belief that drugs should only be taken by a sick person. The unavailability of some TB contacts during the team visit to their various houses and hostility from the TB contacts to the contact tracers during the house-to-house screening of presumptive TB cases due to community stigma associated with TB were reported as other existing gaps in the current TB contact investigations. A few of the respondents also mentioned difficulty in reaching the TB contacts living in hard-to-reach areas due to the deplorable State of the roads, low availability of TPT for TB contacts, and inadequate recording and reporting tools needed for contact tracing by some government officials. One of the STBLM officials specifically said;

*“The issue of the availability of drugs, we do not have enough 3HR, i.e., three months drugs that supposed to be were distributed. So, we are now prioritising what you have in store as being distributed with our normal drug out or commodity in the market based on the high burden. Whereas every facility that handles patients is supposed to have enough drugs so that any contact is identified. We also do not have enough recording and reporting tools for this contact investigation, so certain forms must be used for contact tracing.”*

About half of the community volunteers mentioned that some TB cases do not have a good relationship with their contacts, e.g., neighbours, which makes TB contact investigations a challenge. Also, some of the participants highlighted the fact that some treatment centres are far from the houses of TB patients, and this poses a challenge in the current TB contact investigations. They further said that some TB contacts display a hostile attitude when they are asked specific TB screening questions.

When asked about the challenges that exist in the process of TB contact investigations, almost all the participants mentioned the perceived stigmatisation attached to TB in the community as an inhibitor to the smooth conduct of TB contact investigation because TB patients do not want others around them to be aware of their TB status. Also, participants mentioned low patient education about contact tracing, specifically its importance in TB management, and the fact that most TB patients have little or no information about TB preventive therapy as the notable gap in TB contact investigation. In addition, challenges with regards to the transport mechanisms in getting the TB preventive therapy clients to come to the facility to get their medications, as well as high-cost demands of TB contact investigation, were also stated; this is evident in situations when there is a need for return visits to patient household to conduct TB screening for contacts that were not initially available.

### 3.4. Strategies Currently Employed in Filling the Gaps

Most government officials listed rigorous counselling sessions and advocacy visits to the community and religious leaders (Table A3).

### 3.5. Proposed Strategies for Overcoming the Perceived Challenges

More than half of the government officials mentioned the provision of training to health workers in the area of health-focused counselling and its varied components (awareness counselling, pre and post-test counselling, patient counselling, etc.) to build the capacity of the health workers to offer effective counselling to the TB contacts which will, in turn, promote positive behavioural change towards TB screening and enrollment in TB preventive therapy. One of the STBLM officials specifically said;

*“You know counselling is an ongoing process, a continual process. So, maybe TB-LON 3 can assist in training some of our health workers on counselling, on how to counsel a patient, awareness counselling on pre and post-test counselling, and that area of counselling so that we inculcate it in them so that they would be a good counsellor That one will help us.”*

Other proposed strategies for overcoming the perceived challenges highlighted were the need for development partners like TB LON 3 to provide the GeneXpert machine to the State, donate a source of mobility like motorcycles or vehicles for ease of movement of the TB contacts to the facility for TB screening, recruit more community volunteers and trained them to handle contact tracing and awareness creation with the community, and provision of sufficient incentives and funds to the community volunteers/contact tracers.

The TB-LON 3 project staff identified the provision of TB drugs to cater for long-term dispensation of the drugs to TB patients, engagement of experienced healthcare providers to distribute the drugs (TB treatment and TB prevention), and the need for continuous sensitisation to make the TB program a robust community intervention as the areas of possible partnership to close the gaps in TB contact investigation further.

## 4. Discussion

This study showed insufficient diagnostic tools to cater to the number of samples generated via various demand-creation activities and the turn-over time of test results from within 24 h previously to an average of four days to six weeks currently, which is part of the challenges of TB. A similar study also reported insufficient training of healthcare professionals, staff shortages, stigmatised community, lack of knowledge about TB, illiteracy, inability to persuade patients to submit to sputum tests, and delays in receiving Cartridge Based Nucleic Acid Amplification Test (CBNAAT) results are the difficulties encountered during the conduct of active case finding [10].

The unwillingness of the males in the community to get screened for TB was listed as the major challenge faced with TB programming in this study. However, research reports from other developing countries have testified to an increased notification rate of pulmonary TB in men [11]. There was a relatively high male-to-female ratio of patients with DOTS compared to the gender TB prevalence ratio, possibly reflecting the challenges in engaging men in TB screening and treatment programs [12,13,14,15,16,17].

Our study identified key areas where partnerships with development organisations like TB LON 3 could significantly benefit TB programs. One such area is the provision of essential resources such as the GeneXpert machine and transportation options like motorcycles or vehicles, which could be used for the follow-up of patients and their drug distribution. Studies from Nepal, Uzbekistan, Malaysia, Swaziland, and Zambia have also shown that the cost and availability of transport play critical roles in patients compliance with TB treatment. For example, in Malaysia, non-compliant patients faced higher costs and longer travel times to treatment centres, which impacted their ability to adhere to treatment regimens [18,19,20].

The studies by Gebremariam et al. [21] and Maswanganyi et al. [22] reported that many TB patients believed that insufficient food intake or lack of food contributed to more severe side effects and difficulties in tolerating TB medications. Similarly, Aiyegoro [11] highlighted the issue of non-availability of food due to patients being too weak to work and afford meals, which can lead to nonadherence to TB treatment, often linked to poverty. Our findings align with these previous studies as we also identified the lack of funds for TB patients to eat and visit health facilities for medication refills as a significant challenge. Our study mentioned that recruiting more community volunteers and training them to handle contact tracing and awareness creation with the community will reduce nonadherence of TB cases to drug dosage; this aligns with Iweama et al. [23] which mentioned that health education experts, community health officers, and nurses at the community and health facility levels should implement a more intensive health education program to improve peoples knowledge of TB and its treatment.

In the investigation of contacts of patients with TB, it was reported in the study that the TB contacts are supportive of getting screened and tested for TB; however, the majority who tested negative for TB are unwilling to be placed on TB Preventive Therapy (TPT). The WHO End TB Strategy prioritised TPT among high-risk persons as a key component under Pillar 1. The programmatic management of TPT fits within a larger framework of preventive actions envisaged under Pillars 1 and 2 of the End TB Strategy. It includes screening for TB disease, infection control, prevention and care of HIV and other comorbidities, access to universal health care, social protection, and poverty alleviation [1]. Also, the low availability of TPT drugs in DOT facilities was reported in this study. This is similar to a study in South Africa where it was reported that isoniazid supply shortages only trended towards significance in association with low prescription rates; nearly a quarter of the study participants reported not having TPT medications available when needed [24]. Limited isoniazid supplies have been well-documented as a barrier to TPT implementation [25,26,27]. A 2020 multi-site study in Nigeria identified stockouts of isoniazid as a reason for low TPT uptake and suggested the need to strengthen drug supply logistics to optimise TPT uptake [26]. Isoniazid stockouts serve as a two-fold barrier. First, the apparent absence of medications prevents Health Care Workers (HCWs) from supplying their patients with TPT. Second, a lack of certainty in having an uninterrupted supply of isoniazid generates HCW fear of creating isoniazid resistance [25].

The findings also suggest that contact investigations are generally carried out according to WHO guidelines [28], which recommend screening all close contacts of TB patients for TB disease and Latent TB Infection (LTBI). Contact investigations should be initiated as soon as possible after a TB patient is diagnosed, and all contacts should be screened for TB [29]. The contact investigation team then visits the household of the index patient to identify all household members and any other individuals who may have been in close contact with the index patient. Contacts who test positive for TB are initiated on treatment. In contrast, those who test negative but are at increased risk of developing TB are initiated on preventive therapy, also known as LTBI treatment. Healthcare providers follow up with contacts to ensure they complete their treatment and are monitored for adverse effects [30]. Contact investigation has been proposed as a worthwhile strategy to enhance the early detection of TB cases and reduce transmission in high-incidence localities [31,32].

Stigmatisation attached to TB in the community was identified as an inhibitor to the smooth conduct of TB contact investigation because TB patients do not want others around them to be aware of their TB status. Although the treatment of TB is affected by various biological, cultural, and economic factors, stigma continues to be a major social factor affecting compliance with treatment among patients and influencing their health-seeking behaviours [33,34]. TB is viewed as a stigmatising disease because of its associations with marginalised groups such as the poor [35,36], ethnic minorities [37], low social class [38,39], prisoners and refugees [35,40], and HIV/AIDS patients [37,41,42].

On the proposed strategies for overcoming the perceived challenges, it was identified that the provision of training to health workers in the area of health-focused counselling and its varied components (awareness counselling, pre and post-test counselling, patient counselling, etc.) to build their capacity to offer effective counselling to the TB contacts which will, in turn, will promote positive behavioural change towards TB screening and enrollment in TB preventive therapy. Training health workers is an essential strategy for improving health workers productivity. Poor performance may result from health staff not being sufficient in numbers, not providing care according to standards, and/or not being responsive to the community and patients needs. Apart from training, other influences on the productivity of health workers in tuberculosis control include personal and lifestyle-related factors, living circumstances, adequacy of preparation for work during pre-service education, health-system-related factors such as human resources policy and planning; job satisfaction-related factors such as financial remuneration, working conditions, management capacity and styles, professional advancement and safety at work. These factors constitute a productivity mix, of which tuberculosis training is essential [2].

There are observed strengths in this study; one of these is the ability to interview both the TB patients and their contacts to analyse the problem of TB screening and contact investigation, as well as provide solutions to alleviate the problem. Also, the ward development committee chairs are involved as participants in understanding the community perspective of tuberculosis. However, this does not also come without limitation. Only four out of 33 LGAs were selected in Oyo State, and four out of 30 LGAs in Osun State for this study. Therefore, the generalisation of this finding is limited.

## 5. Conclusions

Conclusively, our findings emphasise the importance of tailored approaches in tuberculosis prevention and control, addressing challenges in testing and contact investigations; this necessitates investments in diagnostic tools, training programs for healthcare personnel, improved access to tuberculosis preventive therapy, and community engagement strategies to enhance cooperation of tuberculosis contacts.

## Data Availability

The data presented in this study are available on request from the corresponding author due to privacy.

## Appendix A

**Table A1:** Sample size for the study.

Osun State				
S/N	LGA	FACILITY	FGD (TBP, HTBC, CV)	KII (DOTS Officers, TBLS, STBLM, WDC Chair)
1	Iwo	Feesu PHC	3	3
2	Ede South	State Hospital Ede	3	3
3	Ife Central	Enuwa PHC	3	3
4	Osogbo	State Hospital, Asubiaro	3	3
		TB-LON 3 project staff		2
		Subtotal	12	14 + 1 (STBLPM)
**Oyo State**				
1	Ibadan North	PHC Sabo	3	3
2	Oyo East	State Hospital, Oyo	3	3
3	Ogbomoso South	PHC Igboyi	3	3
4	Iseyin	General Hospital Iseyin	3	3
		TB-LON 3 project staff		2
		Subtotal	12	14 + 1 (STBLPM)
		Grand Total	24 FGDs	30 KIIs

**Table A2:** Participants Sociodemographic characteristics.

Sociodemographic Variables	KII (N = 30)	FGD (N = 120)
Age *		
15–34	2 (4.1%)	47 (95.9%)
35–60	26 (31.7%)	56 (68.3%)
61 and above	2 (10.5%)	17 (89.5%)
Sex		
Male	21 (24.4%)	65 (75.6%)
Female	9 (14.1%)	55 (85.9%)
Marital status		
Single	7 (16.3)	36 (83.7)
Married	22 (22.4%)	76 (77.6%)
Separated	0 (0.0%)	3 (100.0%)
Divorced	0 (0.0%)	3 (100.0%)
Widow/widower	1 (25.0%)	3 (75.0%)
Religion		
Islam	15 (17.6%)	70 (82.4%)
Christianity	14 (22.6%)	48 (77.4%)
Traditional	1 (33.3%)	2 (66.7%)
Ethnicity		
Yoruba	29 (20.0%)	116 (80.0%)
Igbo	1 (33.3%)	2 (66.7%)
Others	0 (0.0%)	2 (100.0%)
Level of education		
None	0 (0.0%)	12 (100.0%)
Primary	6 (17.6%)	28 (82.4%)
Secondary	5 (8.5%)	54 (91.5%)
OND/NCE	4 (30.8%)	9 (69.2%)
HND/First Degree	12 (46.2%)	14 (53.8%)
Postgraduate degree	3 (50.0%)	3 (50.0%)
Occupation		
Civil or public servant	18 (62.1%)	11 (37.9%)
Trader	3 (8.1%)	34 (91.9%)
Farmer or fisherman	1 (12.5%)	7 (87.5%)
Artisan	0 (0.0%)	26 (100.0%)
Unemployed	0 (0.0%)	12 (100.0%)
Others	8 (21.1%)	30 (78.9%)
Respondent classification		
TB patients	0 (0.0%)	47 (100.0%)
Government Staff	18 (100.0%)	0 (0.0%)
TB contacts	0 (0.0%)	39 (100.0%)
WDC Chair	9 (100.0%)	0 (0.0%)
Community Volunteer	0 (0.0%)	34 (100.0%)
IHVN Staff	3 (100.0%)	0 (0.0%)

* KII Mean age = 48.6 ± 8.5 years; FGD Mean age = 40.4 ± 15.7 years.

**Table A3:** Challenges and Strategies Currently Employed in Filling the Gaps.

Challenges	Strategies
(a) Stigmatisation attached to TB in the community (b) Low patient education about contact tracing (c) Most TB patients have little or no information about TB preventive therapy (d) Transport mechanisms in getting the TB preventive therapy clients to get their medications at the facility (e) High cost of demands of TB contact investigation in in terms of transportation	(a) Rigorous counselling sessions and advocacy visits to the community and religious leaders (b) Creating routine awareness about TB disease in the community (c) Portable digital chest X-ray machine from TB-LON 3 funds has helped increase the testing yield of TB cases in communities and schools (d) Provision of incentive to the TB cases so that they can inform their contacts about the need to be screened for TB (e) Having direct communication between the contacts and the service provider for adequate TB education
